# Practical Nutrition Strategies to Support Basketball Performance during International Short-Term Tournaments: A Narrative Review

**DOI:** 10.3390/nu14224909

**Published:** 2022-11-20

**Authors:** Ozcan Esen, Kazimierz Rozwadowski, Ladislav Cepicka, Tomasz Gabrys, Raci Karayigit

**Affiliations:** 1 Department of Sport, Exercise and Rehabilitation, Northumbria University, Newcastle upon Tyne NE1 8ST, UK; 2 Academic Sports Center, Gdansk University of Technology, 80000 Gdansk, Poland; 3 Department of Physical Education and Sport, Faculty of Education, University of West Bohemia, 30100 Pilsen, Czech Republic; 4 Faculty of Sport Sciences, Ankara University, Gölbaşı, Ankara 06830, Turkey

**Keywords:** sport nutrition, team sport, performance, recovery, dietary supplements

## Abstract

A short-term (e.g., 6 days) basketball tournament is a shorter version of international tournaments, and qualification in it enables participation in international tournaments such as the Olympics and World championships or preparation before major tournaments. Time for recovery between matches is shorter compared with major tournaments, resulting in an accentuated load on players, which can be repeated up to four times within the 6-day competition period. Therefore, nutritional strategies need to focus on faster and adequate recovery after each match as well as optimum fuelling and hydration before and during matches. Travelling can also create additional challenges when preparing and/or applying those nutritional strategies. There are some particular evidence-based sport foods and ergogenic aids that can improve intermittent activity and/or the execution of motor skills, which may facilitate basketball players’ recovery and performance. The present review provides practical nutritional strategies to support short-term basketball tournaments based on players’ physiological needs and current sport nutrition guidelines.

## 1. Introduction

Basketball is a worldwide popular sport that consists of high-intensity intermittent activity patterns. A six-day basketball tournament is a shorter version of international tournaments, and qualification enables participation in international tournaments such as the Olympics and World championships or preparation before major tournaments. Each match consists of four quarters of 10 min (FIBA) or 12 (NBA) min. These times demonstrate actual playing times, but also, the time clock is frequently stopped when the ball is out of play, which increases the total time of the game. The combination of speed, power, agility, skill, endurance, and tactical sense also makes basketball a highly intense sport. Each game consists of repeated sprints, rapid accelerations, and decelerations [1,2], explosive change in directions, jumps, and jostling for position in rebounds [1]. Typical activity patterns of a basketball game have been reported that approximately 1000 discrete movements (e.g., running, jumping, and jostling) are made by changing them every 2 s during the game. Each player does approximately a total of 100 sprints and jostling activities, one every 20 s in actual playing time. In addition, players do more jumps every minute during a match compared with other team sports [3]. Time for recovery between games is shorter (less than 24 h) in 6-day tournaments compared with major tournaments as players need to play four games within 6-day, which results in an increased load on players. This reveals the importance of faster recovery after a match in this type of basketball tournament. Therefore, nutrition strategies are crucial for recovery after a game and preparation for the next one.

The physiological and metabolic demands during a basketball game are very high as high-intensity intermittent bouts are performed for relatively long durations. Recently, it has been reported that a 5–6 km distance is covered with 3.2–6.8 mmol/L (above the threshold) and an average of 85% of maximal heart rate during a 40 min game [3]. Elevated blood lactate concentration during a game indicates that the main fuel source is supplied by glycolysis, whereas heart rate responses indirectly indicate the utilisation of aerobic energy sources [4] Further, a study by Janeira and Maia [1] reported that the covered distances in a single game consisted of 21% of moderate and 20% high speed running and 12% of the number of jumps. Taken together, these findings indicate that players’ energy requirements rely on both aerobic and anaerobic metabolic pathways [2] and that carbohydrate is the primary fuel source for basketball players [5,6]. Considering a number of repeated eccentric muscle contractions are performed during a game [3,7], protein is another crucial nutrient for post-game recovery as it plays a key role in muscle repair and remodelling [8]. It is also important to manage optimal fluid intake pre- during and post-game as there is evidence that basketball players lose a large amount of sweat during a game [9,10].

Several papers in different team sports have reported marked residual fatigue, and reductions in explosive power, and intermittent sprint running during the 3-day tournament [11,12]. These findings can be expected to occur during a short-term basketball tournament. Therefore, players should be fit enough to limit the impact of muscular and physiological fatigue and recover efficiently to play from one game to the next. Traveling (e.g., road trips and air travel) can create an additional challenge when preparing and/or applying nutritional strategies, as it would limit food and drink options and time for their consumption, in addition to causing a negative effect on health and performance [13]. There are some evidence-based functional foods and ergogenic aids that may facilitate players’ recovery, thereby improving performance [14] (e.g., caffeine, nitrate, sodium bicarbonate) given that their potential benefits on muscle contractility, exercise efficiency, muscle pain, and damage. Several reviews have reported nutrition strategies in other team sports, such as soccer [15] and rugby [16], or in-season for basketball [17], but there is no review or report yet about nutrition strategies for a short-term basketball tournament. Therefore, the aim of this review is to examine nutrition strategies and provide a practical application that can be managed during a short-term international tournament.

## 2. Practical Nutritional Strategies Pre-, during, and Post-Games during Tournament

Several studies have investigated the daily dietary intake of basketball players. Whilst daily calorie intake was reported to be relatively high (between 3500 and 5500 kcal/day) [18], daily carbohydrate intake was reported between 4 and 6 g/kg BM in collegiate and/or adolescent basketball players [19,20]. Likewise, it was reported that daily carbohydrate intake was ~5 g/kg a day in elite basketball players [21]. Although this amount just meets the lower range of the current carbohydrate recommendations (5–12 g/kg of body mass) for team sport athletes per day for recovery from moderate to very high-intensity exercise [5,6], a higher range is likely required for game days, especially during a short-term tournament, as performance occurs at very high-intensity. With regard to protein consumption, daily protein intake was reported as 1.5–2 g/kg BM [15,22]. Although this amount is at the recommended level for team sport athletes [8,23], it is important to highlight that the distribution of this amount around the game and throughout the day in addition to the timing of consumption would be crucial to facilitate the repair of the skeletal muscle, bone, and connective tissues during a short-term tournament.

**Figure 1:**
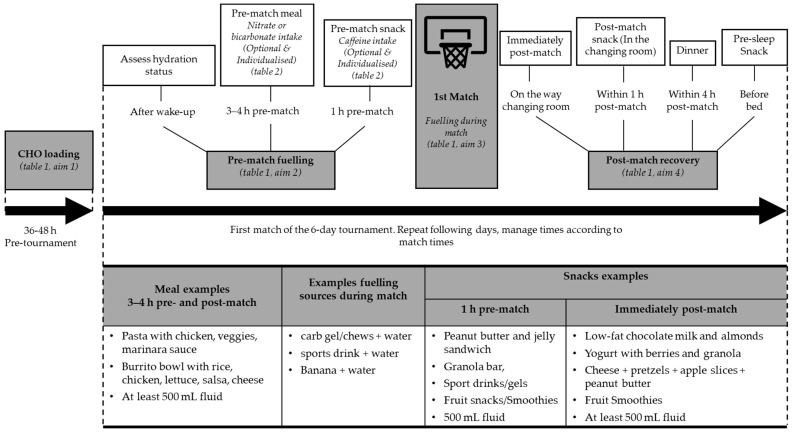
Time guide for optimum nutrition during a typical tournament schedule. Adapted from [16].

The accumulated energy cost over a day in which four matches are played within 6 days is likely to be considerable. Therefore, managing carbohydrate intake, establishing consistent hydration habits, and providing faster recovery are all key nutritional strategies to maximise game-time performance and tournament-long durability for six-day tournaments. These strategies are described in Table 1. An outline of the key nutrition guidelines around a typical tournament schedule is presented in Figure 1.

### 2.1. Fuelling Pre-Games

On match days, energy expenditure and carbohydrate requirements are expected to be higher than on training days [24]. Players can have enough time to recover when they play once a week, however, playing for a few days in a row would decrease their performance due to progressive fluid and glycogen depletion [25]. In these cases, aggressive nutritional strategies should be applied to provide an adequate amount of fluid and carbohydrate [26]. Although there are no specific studies on glycogen depletion on match days in basketball, Bangsbo et al. [27] have reported that marked glycogen depletion occurred after one match in team sport athletes. Therefore, consumption of a large amount of carbohydrate, such as “Carbohydrate-loading”, a day before may benefit basketball players [28]. Given it has been demonstrated that even 36–48 h of taper and high carbohydrate intake increased glycogen storage from ~90 mmol to ~180 mmol/kg wet weight, and thereafter remained stable despite another 2 days of the same conditions [29], “Carbohydrate-loading” a couple of days prior to the tournament may be an effective strategy to maintain performance and facilitate recovery during the tournament.

Matches can be played at different times of day depending on the schedule of the tournament. Therefore, the pre-event mealtime may be taken in the form of breakfast, lunch, dinner, or even a substantial snack. Usually, consumption of an easily digestible meal supplying carbohydrate sources 1–4 h before the match is recommended [23]. Players are generally recommended to consume carbohydrate-rich foods to provide a total of 1–4 g/kg body weight of carbohydrate 3–4 h before, and 1–2 g/kg body weight of carbohydrate 45–60 min before the match [23]. Additionally, the recommendation is to eat a moderate amount of protein and to avoid high-fibre and high-fat foods because they can cause gastrointestinal discomfort and delay digestion, respectively [23]. Supporting this, it was reported that elite basketball players receiving nutritional consultation from certified sports nutritionists have an appropriate diet during match days [30]. The diet consisted of large amounts of whole grains, vegetables, and sodium, but less saturated fat. Whereas increased whole grain and total vegetable intake decreased energy and protein consumption, the range of energy and macronutrient intake (6.8 ± 0.9 g/kg of carbohydrates and 2.2 ± 0.2 g/kg of protein) was still within the recommended range for elite athletes [23].

Being well-hydrated is crucial for performance during a game. Sufficient fluid intake with meals (at least 8–12 before the match) provides a good hydration status on the match day [31]. However, if players have not had enough time or fluid volume to re-establish hydration, aggressive hydration strategies may be applied before the game. Approximately 500 mL of fluid, such as water or sport drinks, is suggested at least 4 h before the game. If players do not produce urine, or urine is dark, approximately 300–500 mL of fluid intake is suggested approximately 2 h before the match [32], which provides sufficient time for urine output of excess fluid prior to the match. Fluid intake with sodium (20–50 mEq.L-1) or small amount of salted snacks may stimulate thirst and, therefore, encourage players to consume fluid [33,34].

### 2.2. Fuel and Fluid during Games

Inadequate fuel and fluid intake may cause fatigue during a match. The glycogen depletion and fluid deficit incurred across a single game could be small, but that may cause players to start the next game with low stored glycogen and be dehydrated in a 6-day style tournament [24,35]. The aim of fluid intake during the match is to prevent excessive dehydration (>2% body weight loss from water deficit). However, the amount and rate of fluid replacement vary according to the individual sweat rate, and match duration, which makes specific fluid and electrolyte replacement recommendations difficult. Therefore, body weight changes during training/competition sessions should be monitored to estimate individual sweat lost during exercise tasks, which provides to make individual fluid replacement programs for each player [31].

While it has been reported that moderate levels of dehydration (~2%) can negatively affect skills and movement patterns, sprint and shooting performance in basketball players [36,37], There is evidence that carbohydrate and/or carbohydrate-electrolyte solution intake during basketball specific exercise improves sprint speed, time to fatigue and cognitive functions and mood state [37,38,39,40], particularly at the last quarter. The current recommendation for carbohydrate intake during a game is 30–60 g/h [5,6,23]. Due to the intermittent and explosive (predominantly anaerobic) nature of basketball, players would benefit from small and frequent amounts of carbohydrate and fluid intake during a match. Indeed, time-outs, between quarters and half-times, and substitutions, provide refuelling and rehydration opportunities that should not be overlooked. Therefore, individual patterns to consume 30–60 g of carbohydrate should be found for players (e.g., 1-2-1 approach: 15 g at the first quarter break-30 g at the half-time break-15 g at the third quarter break).

Sodium should also be added to the fluid to replace salt losses [23]. Despite this being an issue of debate, some players might be more prone to muscle cramps due to salt losses [41]. Therefore, a subjective salt could be added to food and drinks; and a higher sodium version of sports drinks could be used to decrease the risk of cramping for these players [23]. With regard to muscle cramps, another proposed theory is named the altered neuromuscular control theory, which suggests that fatigue and muscle overload result in an imbalance between inhibitory and excitatory impulses to the muscle and subsequently cause muscle cramps [42,43,44]. Based on the altered neuromuscular control theory, pickle juice ingestion has emerged as a growing nutrition intervention to minimise muscle cramps. The acetic acid content of pickle juice, which provides the sour taste, is thought to stimulate oropharyngeal receptors, in turn possibly activating certain neurological sensory inputs, which may mitigate muscle cramps [43,44]. Although further research is required to draw a solid conclusion whether pickle juice reduces muscle cramps, the current recommended dose of pickle juice is between 70 and 100 mL (0.35–0.66 g of acetic acid) for the possible mitigation and management of muscle cramps in practice, training, or competition [43,45]. All these strategies above should be practiced in the training initially. This will provide players to develop and learn their individual fluid intake strategies and tolerance.

While elite-level basketball is usually played indoors where the temperature can be controlled, hot weather and humidity may be still important factors to consider regarding hydration, when there are congested schedules and travel, particularly in warmer areas. In these kinds of situations, since players would produce near-maximal sweat rates, this can cause players to start the game with the risk of hypohydration. Therefore, it would be important (I) to know local environmental conditions beforehand to determine the risk of high sweat rates; (II) to start games well-hydrated; and (III) to increase fluid intake during games in hot and humid environments.

### 2.3. Refuelling–Recovery–Rehydration Post-Game

Recovery nutrition is indispensable for tournaments that have two to three games on successive days since matches are played less than 24 h apart and since sometimes teams will need to change locations between games. These changes provide numerous nutritional challenges due to there being less recovery time in addition to road trip fatigue and substantial depletion of fuel and fluid levels, which must be replenished prior to the next match. Additionally, some muscle damage or injuries may happen due to high-intensity play, which also requires recovery and repair. A well-controlled, real-life tournament study demonstrated that 3 consecutive days of basketball play creates cumulative fatigue [7]. In addition, the process of muscle glycogen restoration can extend until 72 h after a single match in team sports, despite dietary strategies that promote carbohydrate (and protein) replacement [46]. Therefore, firstly, players should aim to restore their depleted muscle and liver glycogen stores immediately post-match as glycogen-synthesising enzymes are most active during the first 30 min and provide muscle glycogen concentrations by 45% higher compared to 2 h after the match [47]. When there is less than a 24 h recovery period between matches, players should target to consume 1–1.2 g/kg of carbohydrate per hour in the first 4 h post-exercise to provide greater glycogen re-synthesis [48]. As an alternative to consuming carbohydrate, the addition of adding protein (0.3–0.4 g/kg) to carbohydrate (0.8–1 g/kg) during recovery can provide a similar glycogen synthesis relative to 1–1.2 g/kg of carbohydrate intake alone [49]. Besides its effect on glycogen synthesis, the post-match carbohydrate-protein mixture would promote muscle protein synthesis [50], which is another fundamental element of recovery.

Basketball players repeat many eccentric muscle contractions during the match as is the nature of intermittent sports, which can trigger muscle damage, and impaired muscle function [51]. The post-match nutrition strategy should target muscle protein synthesis (MPS), because it is key for the muscle repair and remodelling process. Whereas consumption of 0.25 g/kg of protein can optimise the stimulation of MPS, it has recently been reported that higher doses (0.4 g/kg) provide greater MPS [52]. Players should ensure that they consume protein within 30 min post-game and continue to consume every 3–4 h can benefit to maximise MPS, since stimulation of MPS is maximised by 3 h [53]. Animal-based protein (e.g., whey) has a higher leucine content, which is the main trigger for MPS and provides fast digestion and absorption [54]. Given that the diets of some players might be only plant-based, soy protein could be an option for them. Existing evidence shows that consumption of casein protein, which is slowly digested and absorbed, before evening sleep can increase MPS and enhance net protein balance [55,56]. Another key aspect to optimise protein ingestion to benefit recovery is the ingestion of protein before sleep. Since given evening period of sleep (~7–9 h) is the longest period of time wherein individuals do not consume protein. Based on current recommendations [55,56], players can benefit from the consumption of 30–40 g of casein protein prior to sleep to support their recovery.

Rehydration is another important consideration of the recovery process. Aggressive re-hydration strategies could be necessary when players need to play subsequent matches within a short timeframe [34,57]. This is because, inadequate rehydration can impair glycogen restoration and protein synthesis rates [58], sprint capacity [59], and skills [60]. It is reported that athletes need to consume 150% fluid for every 1 kg weight loss during matches [61], which should be consumed within 6 h after the match. Plain water reduces plasma sodium concentration by increasing urine output due to its free electrolyte content [62]. Sodium is a key electrolyte that enhances palatability and stimulates physiological thirst. Therefore, recovery beverages should be high in electrolytes, particularly sodium (50 to 80 mmol/L), for optimum rehydration.

## 3. Nutrition Strategies for Travelling

Travel is a part of the 6-day tournament-style basketball competition and causes additional challenges that impact the nutritional routines of players, as teams may have frequent trips to different areas or cities between games. The issues that are likely to be met during the trip are hydration during the flight, the food available at the destination, catering plans, hygiene standards, and special nutritional needs arising from match goals or from the new environment. Given fluid loss increases during plane, bus, or train travel due to air conditions, airlines, bus, and train companies may be initially contacted about whether to provide additional fluid service. carbohydrate-rich snacks and drinks can be prepared, which will prevent dehydration and keep players fuelled. Hygiene should also be considered in some international destinations. In case of an unsafe local water supply, sealed bottles of water must be prepared. Players need to ensure that bottles are opened in their presence. If there is a buffet, players should choose well-cooked food and avoid salad and raw vegetables when they do not know whether vegetables have been washed in bottled or boiled water.

## 4. Sport Foods and Supplements

Numerous sport foods with special formulations and supplements have been developed to provide energy and nutrients in a form that is easy to consume [62]. Basketball players are often interested in using dietary supplements to meet nutritional targets and reach optimal performance [63] as other athletes in different sports. Players may prefer them for nutritional support before, during, and after a match or as a portable supply of energy and nutrients when real food is unavailable or impractical to consume. Sport nutrition products, such as sports drinks, gels and bars, and liquid meals can be beneficial especially for road trips and during tournaments. The use of supplements, particularly ergogenic aids, is widespread in basketball, but only some supplements may benefit [64], such as caffeine, creatine, nitrate, sodium bicarbonate, and beta-alanine. In the present study, brief information on some particular supplements (Table 2) that could be effective in short-term basketball tournaments following their acute and/or short-term administration is presented according to the field experience of the author, and the reader is directed to the comprehensive review by Maughan et al. [14]. It is also important to note that there are limited previous studies that directly investigated the effects of the different dietary supplements on basketball performance, and thus further studies are required. Additionally, recommendations for the use of dietary supplements are currently generalised for “team sports”.

### 4.1. Caffeine

Supplementation of caffeine (3 mg/kg body mass [BM]) 60 min before exercise was shown to enhance jump height during basketball-specific jumps, the number of body impacts, and overall performance during the basketball game [65]. Similarly, the consumption of caffeinated beverages (including 3 mg/kg BM of caffeine) was reported to improve jump height and total leg muscle power output compared with a placebo [66]. Together, a moderate amount of caffeine (~3–6 mg/kg BM) can provide ergogenic aid for basketball performance. Such doses can be ingested by everyday amounts of coffee, cola drinks, and some sports products (e.g., gels, sports drinks) [64,67,68]. Despite the potential benefits of caffeine ingestion, basketball players must consider their use before games when the game will be played in evening times, as caffeine may result in negative effects on sleep patterns [62].

### 4.2. Bicarbonate

Bicarbonate can buffer excess stomach acidity and have a negative effect on lactic acid. A study by Ansdell and Dekerle [69] reported that supplementation of 0.2 g/kg of sodium bicarbonate 90 and 60 min prior to a basketball game simulation decreased fatigue by protecting contractile elements of the muscle fibres. However, players may have a problem tolerating large doses, which may result in gastrointestinal (GI) problems before matches. Administration of 0.4 g/kg of body mass of sodium bicarbonate for 3 days (split into 3 equal daily doses), enhanced repeated sprint and jump performance without any GI side effects during a simulated basketball exercise [70].

### 4.3. Nitrate/Beetroot Juice

This increases nitric oxide production via an oxygen-independent pathway, which can enhance exercise efficiency/economy and exercise capacity [71]. Existing literature shows that nitrate supplementation may enhance muscle contractility, particularly peak power and time to peak power [72]. There is also evidence that nitrate supplementation enhanced intermittent exercise performance [73,74], sprint performance, and cognitive performance (i.e., reaction time) [75] in recreationally active individuals and trained team-sport athletes. There are a couple of important points to consider when applying nitrate supplementation. Given the effect of nitrate appears less in trained individuals [71], a relatively high amount of nitrate needs to be ingested (~6–12 mmol a day). The ergogenic effect of nitrate supplementation has been reported following both acute and chronic supplementation, multiple-day (3–7 days) supplementation might be appropriate for trained athletes [71,72]. In addition to that, since nitrate supplementation could be ergogenic for both pre-exercise and recovery [71], it can be considered to supplement it more often at intervals during the day (every ~6–8 h). It is well-known that using mouthwash causes blunting the effect of nitrate, and thus, it is suggested athletes avoid using mouthwash when applying nitrate supplementation.

### 4.4. Omega 3

Omega-3 fatty acids, particularly eicosapentaenoic acid (EPA) and docosahexaenoic acid (DHA) are important cell membrane components in the brain and muscles and enhance membrane functions and therefore brain health and muscular performance [76]. Additionally, the anti-inflammatory properties of omega-3 fatty acids may promote faster recovery. While Jouris et al. [77] reported a reduction in eccentric-exercise-induced delayed-onset muscular soreness (DOMS), Gray et al. [78] did not find the same outcomes. Therefore, more research is required for omega-3 and sport recovery.

### 4.5. Anti-Inflammatory (Tart Cherry Juice)

It has been reported that muscle damage and inflammatory markers can be reduced with tart cherry juice consumption after resistance, high-force eccentric, and running exercise [79,80]. In addition, it has been found that faster recovery is more important when there are multiple competitions in a short period [79].

Aforesaid earlier above, this review focused on some particular supplements that can provide benefits for basketball performance with their acute and/or short-term use. However, it is important to knowledge that there are also others that could also benefit basketball performance during short-term tournaments in case of using those in the long term. For example, creatine monohydrate supplementation may improve short-term, high-intensity exercise capacity and multiple-sprint performance via increasing muscle creatine stores and augmenting the rate of phosphocreatine resynthesis [81]. This ergogenic creatine supplementation was reported following a loading protocol, but this can cause 1–2 kg weight gain, as creatine increases muscle water content [81], which may be considered a disadvantage for some team-sport players that involve considerable running and jumps [82]. Therefore, a low-dose protocol that avoids the “loading phase” can be suggested for players to avoid weight gain, which may compromise their power-to-weight ratio before a tournament [83].

In summary, supplement use is widespread in basketball and may enhance performance. Players should prefer evidence-based products and consider the risks because some may even be harmful. Further and more importantly, players should be aware that supplements can cause the antidoping rule violation given that there are reports showing that supplements contain prohibited substances as an undeclared ingredient or contaminant [84,85,86]. Therefore, firstly, players and coaches should be educated to make evidence-based decisions about whether and how to use a specific product. Players should also consult with sport nutritionist, their team doctor, or sport pharmacist before using any dietary supplement. Moreover, players and/or coaches should check if supplements that they consider using have been tested for prohibited substances by third-party auditing.

## 5. Conclusions

Nutrition for basketball players, especially in special tournaments, requires specific knowledge of the physiological demands of the game as well as the associated characteristics of players. However, although basketball is a popular worldwide sport, there are no specific nutrition guidelines. This present review has adapted current guidelines from other team sports with similar physiological stresses. Future studies need to investigate the relationships between the physiological demands of basketball and nutritional strategies to optimise basketball performance. Furthermore, players’ current nutrition should be researched in order to provide the targeted individual approach for optimal fuelling, hydration, and recovery during training and games. The present review provides practical nutritional strategies to support short-term basketball tournaments based on its physiological demands and current sport nutrition guidelines.

## Figures and Tables

**Table 1:** Practical nutrition strategies for optimum performance during international short-term basketball tournaments. Adapted from [23].

Aim	Guidelines	Practical Applications
Optimum glycogen stores before tournament	7–12 g per kg for 24–48 h before tournament (‘Carbohydrate loading’) Optimise hydration status (at least 8–12 h before) Monitor urine colour night before and 2–4 h before matches (pale colour)	Consume carbohydrate-rich foods and drinks at each meal and snack. Adding 2–4 extra carbohydrate-rich snacks alongside three main meals could be more appropriate instead increase the number of carbohydrates in main meals. Moderate protein intake and low fibre and fat intake. Arrange snack intake around hotel catering (Three daily meals). Plan pre- and post-training snacks. Ingest fluid at every meal and snack. Individualise fluid bottles for players.
2. Pre-game fuelling	Carnohydrate-rich (1–4 g per kg) pre-match meal 3–4 h and snack 1–2 h before matches 300–600 mL fluid intake 3–4 h before the match 200–250 mL fluid intake 1–2 h before the match 200–250 mL water can be consumed 15 min before the match	Familiar and suitable pre-match meals and snacks. Try to arrange hotel catering before tournament. Consume carbohydrate-rich foods such as bread, potatoes, rice, pasta, and fruits for building your pregame meal. Balance the rest of your plate with lean, low-fat proteins and low-fat dairy. Include fluids with your meal to begin hydrating for the game. Provide different fluid options to encourage voluntary consumption. At least two cups (500 mL) of water, sport drink, or 100 percent juice are good choices. If you are prone to muscle cramps, eat salty foods such as pretzels and crackers; or consume sport drinks. Check your urine colour after waking up, and 4, 2, and 1 h pre-game. If the colour is not pale, drink fluid between 0.25 and 1 L depending on colour.
3. During game	Mouth rinse with carbohydrate fluids or, small amounts of carbohydrates (30–60 g/h)	Players who play for many minutes should consume small portions of carbohydrate food at halftime to reload energy for the second half. Some good options are sport drinks, sport bars, or sport gels. Experiment with different strategies during training, which will provide to find what works best for each individual player during the game. Use breaks (time-outs and substitutions) in the game to hydrate intentionally.
4. Post-match recovery	~1 g per kg immediately after match (within 5–30 min) 1 g per h for the first 4 h of recovery (ideally at 1st, 2nd, and 4th h) 0.3–0.4 g/kg of protein within 30 min after match immediately after match. 1.5 L of fluid for each kg of weight loss aim to consume the target volume over the next 2–4 h 30–40 g of protein before bed	Plan recovery snacks or drinks for immediate post-match intake. For example, 3:1 carbohydrate and protein, respectively on the way to changing room, Choosing a bar or meal replacement drink will likely provide adequate protein to meet your body’s recovery needs, but not adequate carbohydrates. Eat additional recovery fuelling and hydration meal or snack within two hours post-match to continue the recovery process. It can be provided in the change room (1 h post-match) and/or on the coach (if away; 2 h post-match). Within 4–5 h, carbohydrate-rich dinner at the hotel.
5. Travelling		Investigate food and dining options before traveling. Review hotels’ or/and restaurants’ menus and the availability of in-room refrigeration. Pack snacks and foods (oatmeal, granola, peanut butter, bagels). Just in case, plan for snacks or mini-meals based on the competition schedule. Investigate grocery stores that are near hotels and competition sites. Choose restaurants where sandwiches, light meals, and low-fat options are available. Call ahead to reserve areas, and review menus online for competition-site deliveries.

**Table 2:** Evidence-based [14] use of dietary supplements to benefit match outcomes.

Supplement	Aim	Recommended Protocol of Use		Comments
		Amount	Timing	
Caffeine	Performance	3–6 mg/kg	1 h pre-match	Different sources such as coffee, coke, sport drinks, and gels
Sodium bicarbonate	Performance	0.2–0.4 g/kg	2–2.5 h pre-match	In case of GI upset, daily smaller doses a couple of day pre-tournament
Nitrate/beetroot juice	Performance/recovery	6–12 mmol	2.5–3 h pre-match	5–7 days of pre-tournament supplementation may benefit better.
Omega 3-fatty acids	Recovery	2 g/day	Before bed	Faster muscle recovery period.
Anti-inflammatory Supplements: Tart cherry juice	Recovery	250–300 mL(30 mL. if concentrate)	Post-matchBefore bed	Faster muscle recovery period.

## Data Availability

Not Applicable.
